# Hypoxia induces pulmonary fibroblast proliferation through NFAT signaling

**DOI:** 10.1038/s41598-018-21073-x

**Published:** 2018-02-09

**Authors:** Lakmini Kumari Senavirathna, Chaoqun Huang, Xiaoyun Yang, Maria Cristina Munteanu, Roshini Sathiaseelan, Dao Xu, Craig A. Henke, Lin Liu

**Affiliations:** 1 Oklahoma Center for Respiratory and Infectious Diseases, Oklahoma State University, Stillwater, OK 74078 USA; 2 Department of Physiological Sciences, Lundberg-Kienlen Lung Biology and Toxicology Laboratory, Oklahoma State University, Stillwater, OK 74078 USA; 3 Department of Medicine, Division of Pulmonary, Allergy, Critical Care and Sleep Medicine, University of Minnesota, Minneapolis, Minnesota USA

## Abstract

Idiopathic pulmonary fibrosis (IPF) is a chronic, progressive and typically fatal lung disease with a very low survival rate. Excess accumulation of fibroblasts, myofibroblasts and extracellular matrix creates hypoxic conditions within the lungs, causing asphyxiation. Hypoxia is, therefore, one of the prominent features of IPF. However, there have been few studies concerning the effects of hypoxia on pulmonary fibroblasts. In this study, we investigated the molecular mechanisms of hypoxia-induced lung fibroblast proliferation. Hypoxia increased the proliferation of normal human pulmonary fibroblasts and IPF fibroblasts after exposure for 3–6 days. Cell cycle analysis demonstrated that hypoxia promoted the G1/S phase transition. Hypoxia downregulated cyclin D1 and A2 levels, while it upregulated cyclin E1 protein levels. However, hypoxia had no effect on the protein expression levels of cyclin-dependent kinase 2, 4, and 6. Chemical inhibition of hypoxia-inducible factor (HIF)-2 reduced hypoxia-induced fibroblast proliferation. Moreover, silencing of Nuclear Factor Activated T cell (NFAT) c2 attenuated the hypoxia-mediated fibroblasts proliferation. Hypoxia also induced the nuclear translocation of NFATc2, as determined by immunofluorescence staining. NFAT reporter assays showed that hypoxia-induced NFAT signaling activation is dependent on HIF-2, but not HIF-1. Furthermore, the inhibition or silencing of HIF-2, but not HIF-1, reduced the hypoxia-mediated NFATc2 nuclear translocation. Our studies suggest that hypoxia induces the proliferation of human pulmonary fibroblasts through NFAT signaling and HIF-2.

## Introduction

Idiopathic pulmonary fibrosis (IPF) is a chronic and progressive interstitial lung disease, and only limited treatments available. In IPF, pulmonary fibroblasts proliferate rapidly and differentiate into myofibroblasts, resulting in the production of excessive amounts of extracellular matrix proteins and formation of a fibrotic milieu. These consequences destroy the lung architecture and disturb normal lung function^1,2^.

Hypoxia, also known as low oxygen tension, is a prominent feature in many pathological disorders, including respiratory disease, heart disease and cancers^3^. Hypoxia also contributes to the pathogenesis of fibrotic diseases^4–6^. Hypoxia regulates the expression of many genes through hypoxia-inducible factors (HIFs)^7^. There are three isotypes, HIF1, HIF2 and HIF3. Each isoform is composed of two subunits, alpha (α) and beta (β). The structure and functions of HIF-1α and HIF-2α are closely related, while HIF-3α is more distantly related. The HIF-β subunit is constitutively expressed, and the HIF-α subunit is sensitive to oxygen levels. When oxygen concentrations are low, proline residues in the amino- and carboxyl-terminal oxygen-dependent degradation domains (NODDD and CODDD, respectively) of the HIF-α subunit are not hydroxylated since proline hydroxylase is inactive, and the HIF-α subunit avoids proteasomal degradation^8^. The stabilized HIF-α is then translocated to the nucleus, where it binds to the HIF-β subunit and initiates gene transcription^3^.

HIFs regulate the expression of several genes, such as c-Myc, involved in cell proliferation^9^. Several studies have demonstrated the contributions of HIF-1 and HIF-2 to the pathogenesis of pulmonary fibrosis^10–12^. HIF-1 induction has been suggested to be an early event in the pathogenesis of IPF since the upregulation of HIF-1 has been found in histologically normal areas of IPF lungs. The downstream target genes of HIF-1, such as *vegfa*, *cxcl12*, and *pgk1*, were upregulated in the lungs of a bleomycin-induced murine fibrosis model^10^. Conditional knockout of HIF-1α in alveolar epithelial cells of mice reduces bleomycin-induced pulmonary fibrosis^12^. Despite these advances, there have been few reported studies regarding the effects of hypoxia on pulmonary fibroblast proliferation. In one study, hypoxia was demonstrated to increase IPF fibroblast proliferation via miR-210-mediated regulation of the c-Myc inhibitor MNT^11^. In another study, moderate hypoxia (2% O_2_) was found to increase the proliferation of normal human lung fibroblasts through the p21 pathway in a p53-independent manner^13^. However, severe hypoxia (0.1% O_2_) actually arrested the cell cycle through the p53-p21 pathway.

It is known that hypoxia depolarizes the cell membrane and opens voltage-gated calcium channels, leading to calcium influx^14^. An increase in intracellular Ca^2+^ can activate the phosphatase calcineurin, which dephosphorylates nuclear factor of activated T cells (NFAT). Dephosphorylated NFAT is then translocated into the nucleus, where it induces the expression of many genes involved in different cellular pathways^15–17^. NFAT has several different isoforms, including NFAT-1 (NFATc2), NFAT-2 (NFATc1), NFAT-3 (NFATc4), NFAT-4 (NFATc3) and NFAT-5. Whereas NFAT-5 is regulated by osmotic stress, NFAT-1, 2, 3, and 4 are regulated by the calcium/calcineurin pathway. Though NFAT has been widely studied in immune cell activation, these molecules are also involved in the genetic regulation of many cellular processes, including the cell cycle, development, differentiation and angiogenesis^18–20^. Additionally, activation of NFAT signaling has been demonstrated in pulmonary vascular smooth muscle cells (PASMCs) of mice^21^ under hypoxic conditions, suggesting the involvement of NFATc3 in pulmonary arterial remodeling and pulmonary arterial hypertension, respectively.

We hypothesize that hypoxia induces human pulmonary fibroblast proliferation by activating NFAT signaling via HIF-2α. The objectives of this study are to determine 1) the effects of hypoxia on pulmonary fibroblast proliferation, 2) whether NFAT signaling is involved in hypoxia-induced pulmonary fibroblast proliferation, and 3) how hypoxia activates NFAT signaling.

## Results

### Hypoxia induces cell proliferation in normal and IPF human pulmonary fibroblasts

**Figure 1:**
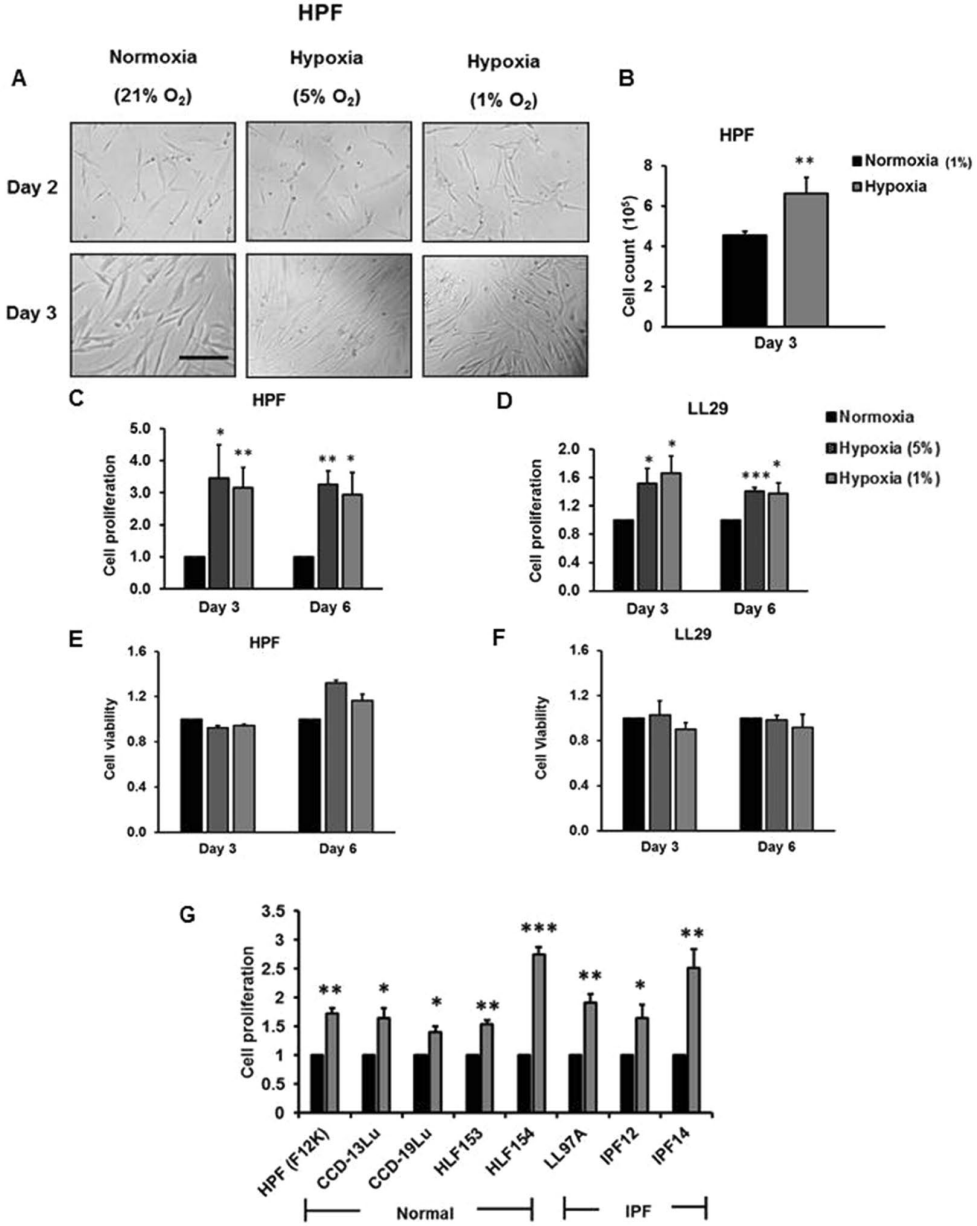
Hypoxia induces pulmonary fibroblast proliferation. Normal human pulmonary fibroblasts (HPFs) and LL29 IPF fibroblasts were exposed to normoxia (21% O_2_) or hypoxia (5% or 1% O_2_) for 2–6 days. (**A**) Bright field imaging. Scale bar: 100 µm. (**B**) Cell count. (**C**,**D**) Cell proliferation by BrdU assay. HPF and LL29 cells were incubated with BrdU for 3 hrs. Data were normalized to normoxia. The absorbance for BrdU at normoxia was 0.08 ± 0.002 (day 3) and 0.09 ± 0.009 (day 6) compared to 0.09 ± 0.004 (day 3) and 0.10 ± 0.006 (day 6) for HPF and LL29 cells, respectively. (**E**,**F**) Cell viability by MTT assay. The absorbance for MTT at normoxia was 0.23 ± 0.0796 (day 3) and 0.30 ± 0.091 (day 6) compared to 0.07 ± 0.016 (day 3) and 0.28 ± 0.097 (day 6) for HPF and LL29 cells, respectively. (**G**) Additional normal human pulmonary fibroblasts [HPF (with F12K medium), CCD-13Lu, CCD-19Lu, HPF153 and HLF154] and IPF fibroblasts (LL97A, IPF12 and IPF14) were subjected to normoxia and hypoxia (1% O_2_) for 3 days, and cell proliferation assessed by BrdU assay. Cells were incubated with BrdU for 12 hrs. The absorbance values for BrdU at normoxia were 0.15 ± 0.006 (HPF with F12K medium), 0.09 ± 0.004 (CCD-13Lu), 0.18 ± 0.008 (CCD-19Lu), 0.28 ± 0.003 (HLF153), 0.14 ± 0.008 (HLF154), 0.11 ± 0.004 (LL97A), 0.05 ± 0.003 (IPF12) and 0.10 ± 0.01 (IPF14); values represent means ± SE. *p < 0.05, **p < 0.01, ***p < 0.001 vs. normoxia. n = 3 independent experiments.

To determine the effects of hypoxia on the proliferation of human lung fibroblasts, microscopic observations, cell counting with trypan blue staining and bromodeoxyuridine (BrdU) assays were performed. Using normal human pulmonary fibroblasts (HPFs) cultured in Fibroblast Growth Medium 2 containing 2% fetal bovine serum (FBS), 1 ng/ml basic fibroblast growth factor, and 5 µg/ml insulin provided by the Manufacturer (PromoCell), an increased number of cells under hypoxic conditions were observed compared to normoxic conditions at culture day 2 and day 3 (Fig. 1A). This increase was confirmed by direct cell counting (Fig. 1B). Cell proliferation, determined by BrdU incorporation assay, was increased in cells grown in hypoxic conditions (5% and 1% O_2_) for 3 and 6 days, and there were no significant differences in fold-increases between day 3 and day 6 (Fig. 1C). Similar results were observed with IPF fibroblasts (LL29) cultured in F12K medium containing 10% fetal bovine serum. However, fold-increases in IPF fibroblasts induced by hypoxia were smaller than those in normal HPFs (Fig. 1D). Cell viability was not affected by hypoxic conditions in both cell types, as assessed by MTT assay (Fig. 1E,F).

To determine whether the culture medium has any effects on hypoxia-induced cell proliferation, we cultured HPFs in F12K medium containing 10% FBS and performed BrdU assays. Our results showed that hypoxia still increased the cell proliferation, although the fold-change in the F12K medium was smaller compared to that in the manufacturer’s medium (Fig. 1G). Furthermore, we tested 7 additional fibroblast lines: four normal human pulmonary fibroblasts (CCD-13Lu, CCD-19Lu, HLF153 and HPF154) and three IPF fibroblasts (LL97A, IPF12 and IPF14). These fibroblasts were cultured in F12K medium containing 10% FBS. Cell proliferation of all fibroblast lines was increased by 3-day hypoxic exposure (Fig. 1G). In all of the studies reported in this paper except Fig. 1G, HPF cells were cultured in in Fibroblast Growth Medium 2 containing 2% fetal bovine serum (FBS), 1 ng/ml basic fibroblast growth factor, and 5 µg/ml insulin as recommended by the manufacturer. In Fig. 1G, the proliferative response was measured in HPF cells cultured in F12K media to ensure that the responses could be compared across all control and IPF fibroblast cultures, all of which were maintained in F12K media.

### Hypoxia promotes G1/S transition and alters cell cycle protein expression in HPFs

**Figure 2:**
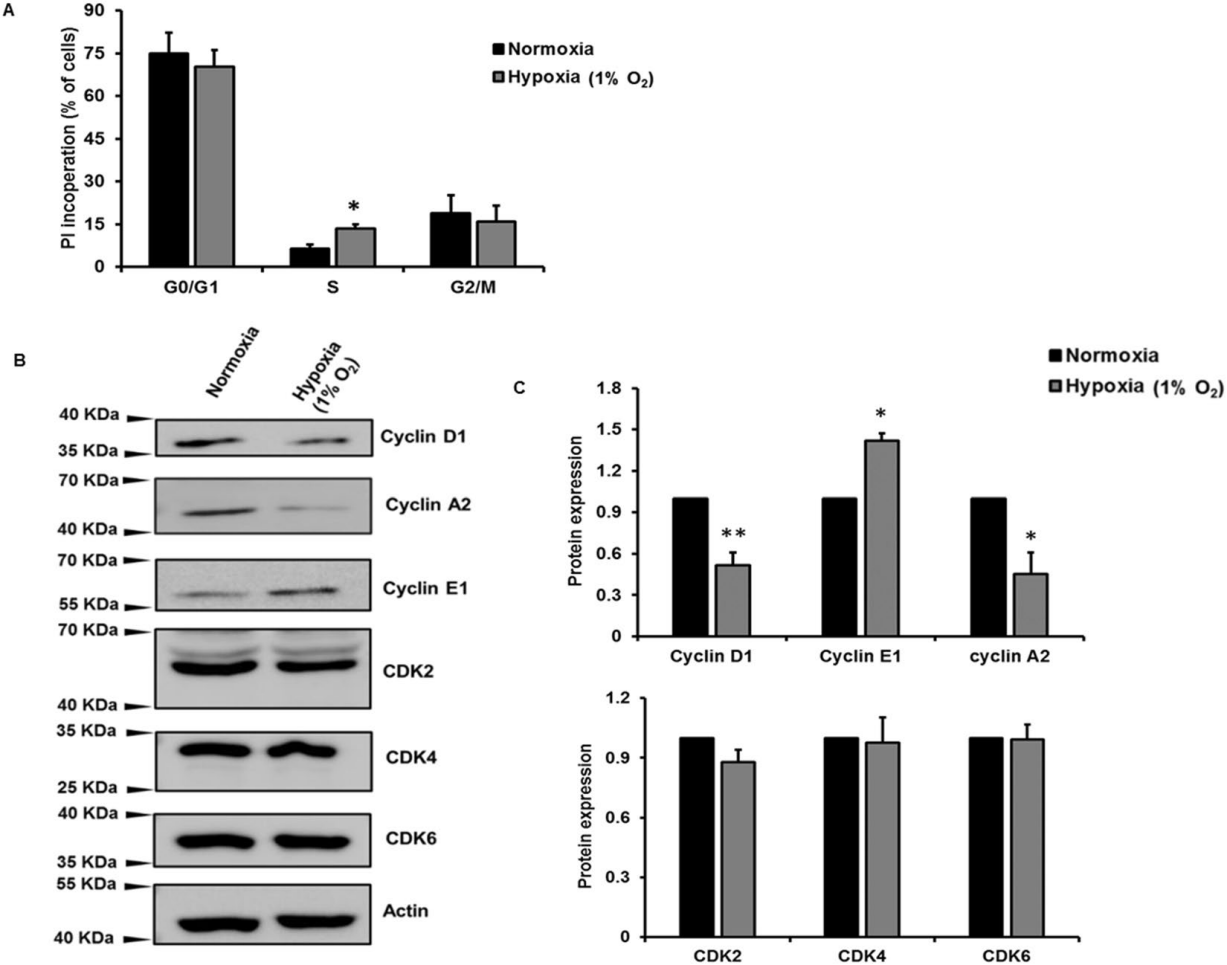
Hypoxia promotes G1/S phase transition and alters cell cycle protein expression in HPFs. HPF cells treated with normoxia and hypoxia (1% O_2_) for 3 days. (**A**) FACS analysis measuring the incorporation of propidium iodide (PI) in proliferating cells; the percentage of PI-incorporating cells in each cell cycle phase is represented. (**B**) Cyclin D1/ A2/ E1 and CDK 2/4/6 protein expression levels were determined by Western blot. (**C**) Cyclin and CDK protein levels were analyzed by ImageJ and normalized to the protein loading control β-actin. The resulted data were then normalized to normoxia group. Data represent means ± SE. *p < 0.05, **p < 0.01, vs. normoxic group. n = 3 independent experiments.

To determine which phase of the cell cycle is affected, fluorescence-activated cell sorting (FACS) cell cycle analysis was performed on HPFs exposed to hypoxia for 3 days using a Cell Cycle Phase Determination kit. The results showed that the number of cells with propidium iodide incorporation in the S phase of the cell cycle was higher in HPFs exposed to hypoxic conditions compared to normoxic conditions (Fig. 2A). These results suggest that hypoxia increases the G1/S phase transition. To determine which cell cycle proteins are affected by hypoxic treatment, levels of cyclins and cyclin-dependent kinases (CDKs) were assessed by Western blot analysis. The results showed that cyclin D1, which is involved in G1 phase, was downregulated (Fig. 2B,C). Cyclin E1, the prominent cyclin in the G1/S phase transition, was upregulated. Cyclin A2, which promotes the transition of the G2/M phase, was downregulated in HPFs exposed to hypoxia. CDK2, CDK4 and CDK6 protein levels were not affected by hypoxia.

### HIF-2α is involved in hypoxia-mediated pulmonary fibroblast proliferation

**Figure 3:**
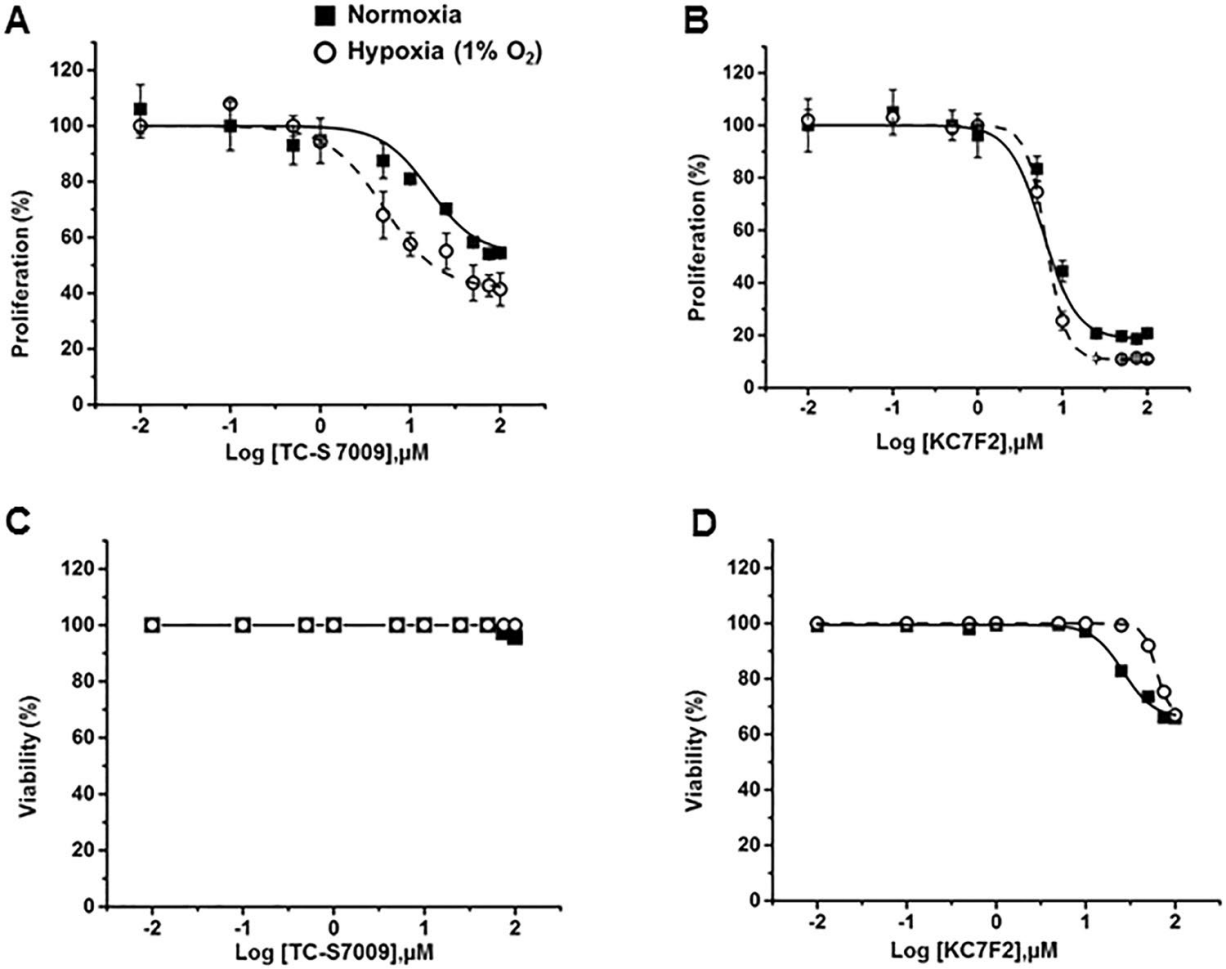
Effects of HIF inhibitors on HPF cell proliferation. HPFs were treated with the HIF-1α inhibitor KC7F2 or the HIF-2α inhibitor TC-S 7009 and exposed to normoxia and hypoxia (1% O_2_) for 3 days. (**A**,**B**) Cell proliferation as determined by BrdU assay. Cells were incubated with BrdU for 12 hrs. (**C**,**D**) Cell viability as determined by LDH assay. Data were expressed as a percentage of control (0 µM inhibitor) for each oxygen condition. The absorbance of control at normoxia for proliferation was 0.39 ± 0.06 (n = 3) and for hypoxia was 0.66 ± 0.16 (n = 3). The viability (%) was calculated as 100% -cytotoxicity %. Cytotoxicity % = [(LDH activity of sample -Spontaneous LDH activity)/(Maximum LDH activity -Spontaneous LDH activity)] × 100. The absorbance of control at normoxia for LDH activity was 0.17 (n = 2) and for hypoxia was 0.16 (n = 2). Maximum and spontaneous LDH activity values for normoxia were 0.26 and 0.17 (n = 2), respectively. Maximum and spontaneous LDH activity values for hypoxia were 0.27 and 0.17 (n = 2), respectively. Values represent means ± SE for proliferation and means for viability.

Hypoxia induces HIF-1α and HIF-2α protein expression, and knockdown of HIF-2α but not HIF-1α inhibits proliferation of IPF fibroblasts^11^. To determine which isoform is involved in normal pulmonary fibroblast proliferation, we inhibited HIFs with isoform-specific inhibitors and assessed their effects on hypoxia-induced proliferation in HPFs. The HIF-2α inhibitor TC-S 7009 inhibits the transcriptional activity of HIF-2 by binding to HIF-2α and interrupting the HIF-2α and β heterodimerization^22^ and does not have effects on HIF-1 activity^23^. The HIF-1α inhibitor KC7F2 has been shown to reduce HIF-1α protein synthesis^24^. We treated HPFs with different concentrations of TC-S 7009 or KC7F2 and exposed them to normoxic or hypoxic conditions for 3 days. HPF cells treated with the HIF-2α inhibitor TC-S 7009 showed greater inhibition of cell proliferation in hypoxic conditions than that in normoxic conditions (Fig. 3A). In contrast, treatment with the HIF-1α inhibitor KC7F2 did not show any differential effects on HPF proliferation between normoxic and hypoxic conditions (Fig. 3B). Lactate dehydrogenase (LDH) assays revealed that TC-S 7009 had no toxic effects at the concentrations tested and that KC7F2 did not affect cell viability at concentrations up to 10 μM (Fig. 3C,D). These results were consistent with previous HIF knockdown studies in IPF fibroblasts^11^.

### Hypoxia activates NFATc2 signaling

**Figure 4:**
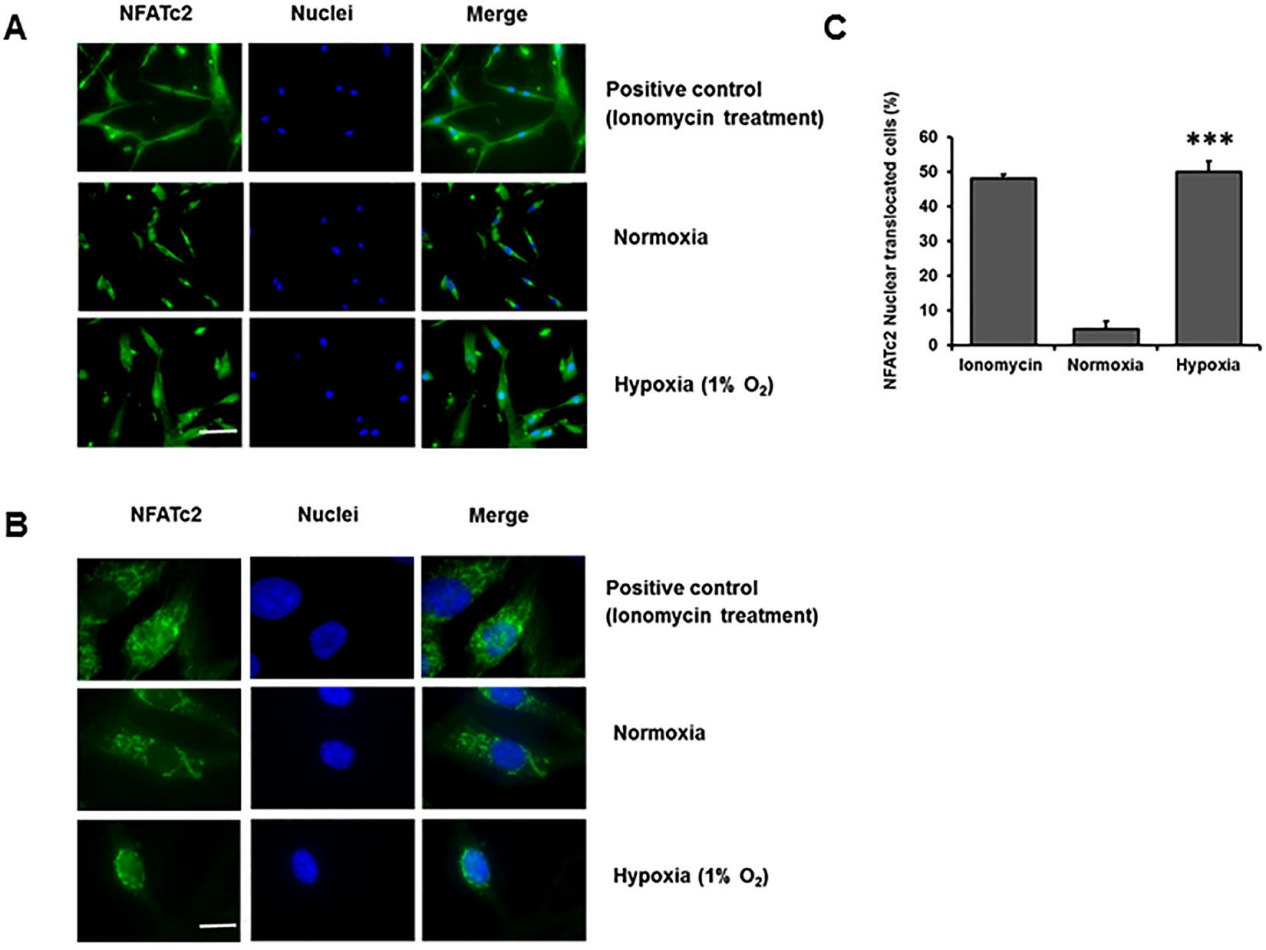
Hypoxia induces NFATc2 nuclear translocation. (**A**) Immunofluorescence staining of NFATc2 in HPFs exposed to normoxia and hypoxia (1% O_2_) for 3 days. Scale Bar: 50 µm. (**B**) Immunofluorescence staining of NFATc2 at higher magnification. Scale Bar: 10 µm. (**C**) Percentages of NFATc2 nuclear-translocated cells were determined by counting the cells with an NFATc2 nuclear localization signal compared to the total number of cells. Data represent means ± SE. ***p < 0.001 vs. normoxic group. n = 3 independent experiments.

Several studies have demonstrated that NFAT signaling is involved in the cell proliferation of various cell types, including PAMSCs^17,25–27^, neural stem cells^28^, β cells, cancer^29^ and lymphocytes^30^. We have recently shown that NFATc2 is required for Wnt5a-mediated fibroblast proliferation^31^. We hypothesized that hypoxia-mediated fibroblast proliferation may be due to the activation of NFATc2 signaling by hypoxia. To test this hypothesis, we determined whether hypoxia could activate NFAT signaling via NFATc2 nuclear translocation. Immunofluorescence staining of HPFs exposed to hypoxia was performed to evaluate the cytoplasmic and nuclear location of NFATc2. Ionomycin, a known activator of NFAT signaling^32^, was used as a positive control. Under normoxic conditions, only 5 ± 2% of NFATc2 was located in the nuclei (Fig. 4A–C). Ionomycin treatment increased the number of cells with NFATc2 nuclear localization to 48 ± 1%. Hypoxia exposure resulted in a significantly higher number of NFATc2 nuclear translocated cells (50 ± 3%) compared to normoxia.

**Figure 5:**
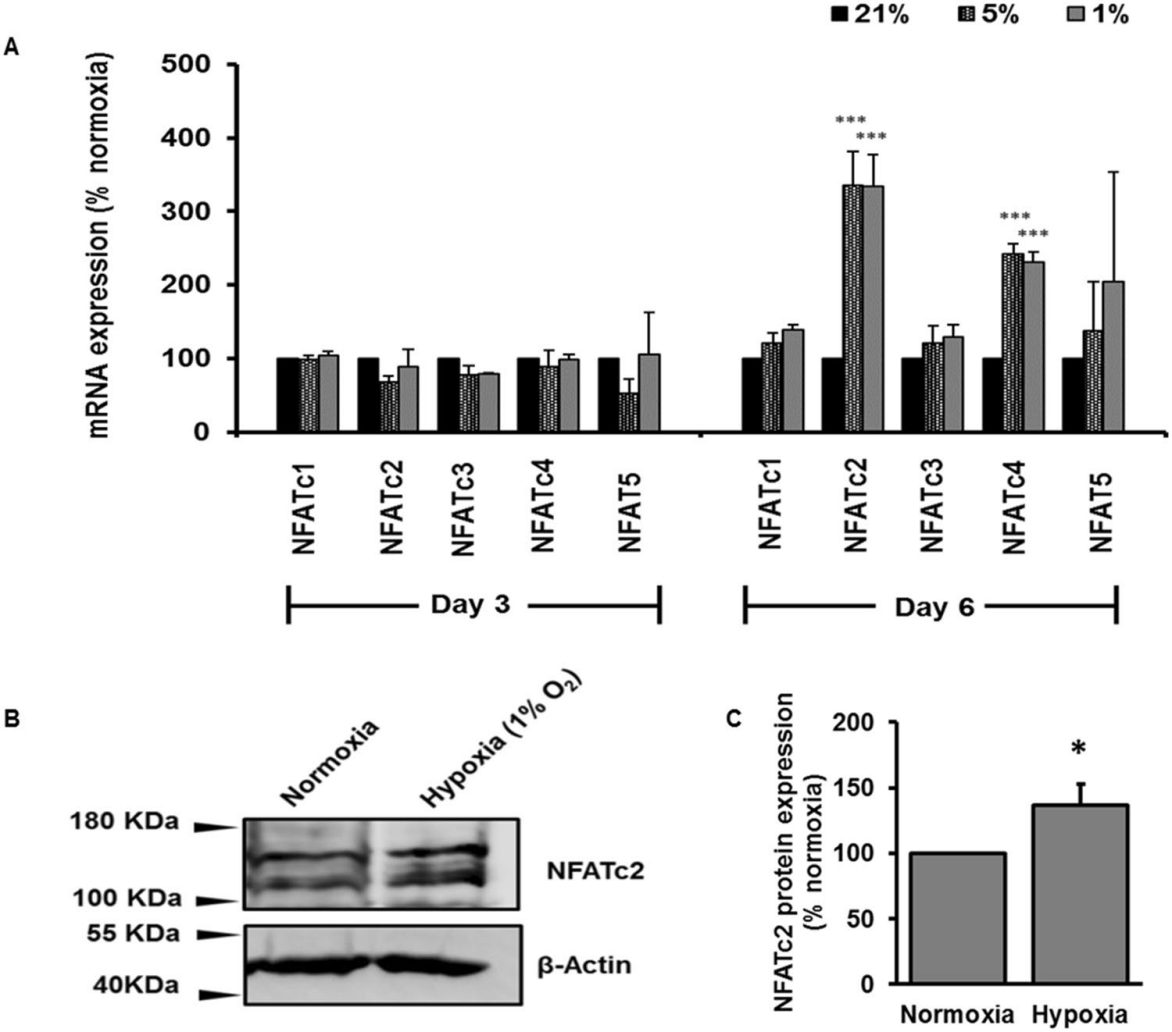
Effect of hypoxia on NFAT mRNA and protein expression. HPFs were exposed to normoxia (21% O_2_) or hypoxia (5% or 1% O_2_) for 3 and 6 days. (**A**) mRNA expression levels of NFAT isoforms were determined by real-time PCR and normalized to β-actin. (**B**,**C**) NFATc2 protein expression levels were determined by Western blot, analyzed by ImageJ and normalized to the protein loading control β-actin. The resulted data were then normalized to normoxia group and represented as a percentage change relative to normoxia. Data represents means ± SE. *p < 0.05, *p < 0.01, ***p < 0.001 vs. normoxic group. n = 3 independent experiments.

We further examined the effects of hypoxia on NFAT expression. We exposed HPF cells to normoxia and hypoxia (5% or 1% O_2_, respectively) for 3 and 6 days and determined mRNA expression levels of NFAT isoforms. Hypoxia had no effect on the mRNA levels of all NFAT isoforms (NFATc1-4 and NFAT5) at day 3 (Fig. 5A). However, hypoxia increased the mRNA expression levels of NFATc2 and NFATc4 by day 6. The induction of mRNA expression of NFATc2 by hypoxia was greater than that of NFATc4. NFATc2 protein expression was also significantly increased by hypoxia at day 6 in HPFs (Fig. 5B,C).

### Hypoxia-mediated activation of NFATc2 signaling occurs via HIF-2α

**Figure 6:**
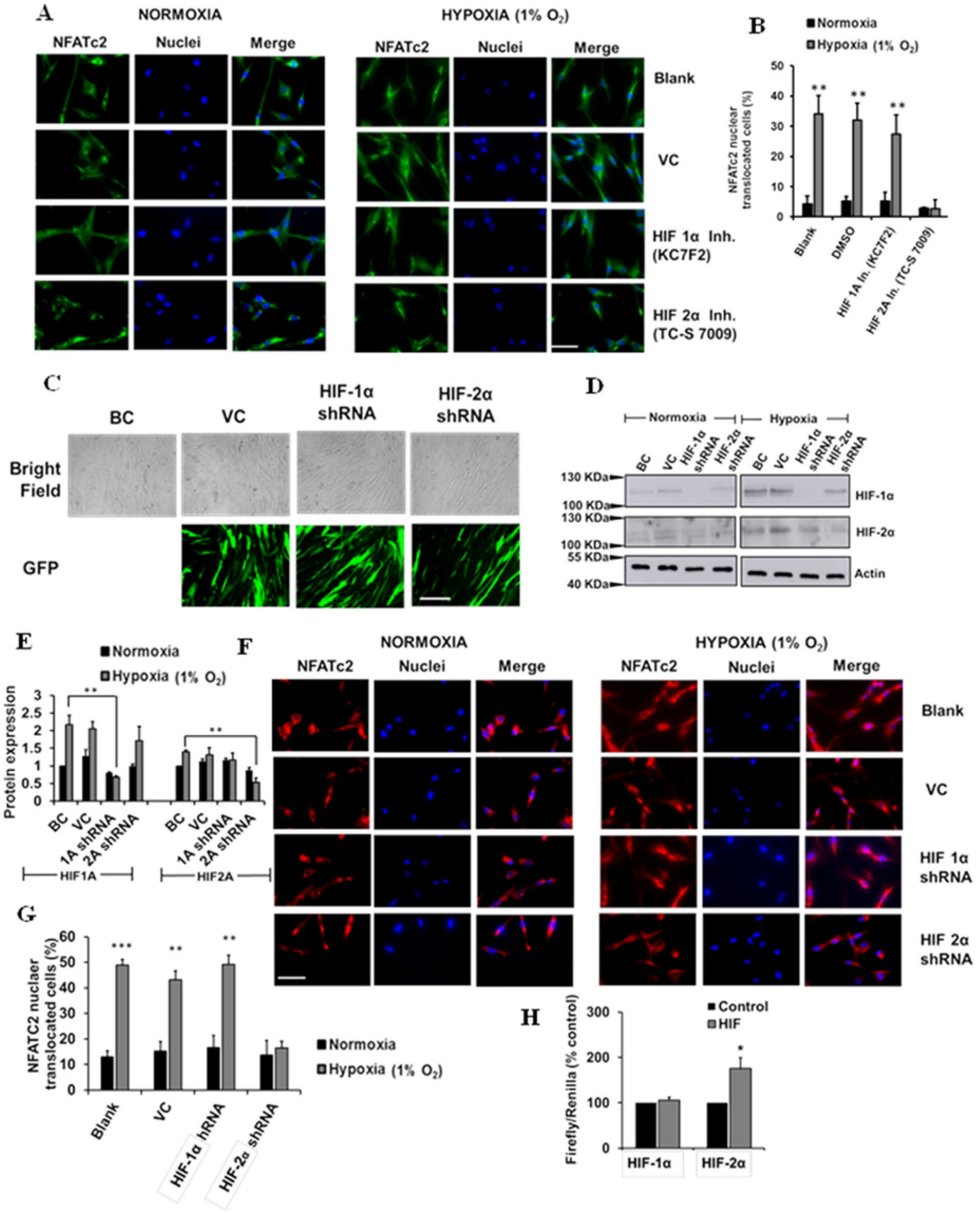
Inhibition and silencing of HIF-2α reduces hypoxia-mediated NFATc2 nuclear translocation. (**A**) Immunofluorescence staining of NFATc2 in HPFs treated with HIF-1α and HIF-2α inhibitors (KC7F2, 10 µM and TC-S 7009, 50 µM, respectively) and exposed to normoxia and hypoxia (1% O_2_) for 3 days. Scale Bar: 50 µm. (**B**) Percentages of NFATc2 nuclear-translocated cells were determined by counting the cells with NFATc2 nuclear localization signals compared to the total number of cells. (**C**) Lentiviral infection efficiency. BC = Blank control, VC = Vector control. (**D**) Western blot showing HIF-1α and HIF-2α silencing efficiency. (**E**) Quantitative representation of protein expression of HIF-1α and HIF-2α protein expression with HIF silencing. (**F**) Immunofluorescence staining of NFATc2 in HPFs treated with shRNA lentiviral constructs (MOI 100) of HIF-1α and HIF-2α and exposed to normoxia and hypoxia (1% O_2_) for 3 days. Scale Bar: 50 µm. (**G**) Percentages of NFATc2 nuclear-translocated cells were determined by counting the cells with NFATc2 nuclear localization signals compared to the total number of cells. (**H**) HEK 293Ts were co-transfected with an NFAT reporter plasmid and HIF-1α or HIF-2α expression vector for 24 hrs. The reporter activities were measured as the ratio of Firefly/Renilla luciferase activities. Data represent means ± SE. *p < 0.05, **p < 0.01, ***p < 0.001 vs. normoxia or control group. n = 3 independent experiments.

Since HIF-2α is the isoform involved in hypoxia-mediated fibroblast proliferation (Fig. 3), we next examined whether HIF-2α participated in hypoxia-mediated activation of NFATc2 signaling. We inhibited HIF-1α or HIF-2α using specific inhibitors or knocked down HIF-1α or HIF-2α using lentiviral shRNAs containing green fluorescence protein (GFP), and determined their effects on NFATc2 nuclear translocation in HPFs. While the HIF-1α inhibitor KC7F2 had no effects on NFATc2 nuclear translocation, the HIF-2α inhibitor TC-S 7009 almost completely inhibited the hypoxia-induced NFATc2 nuclear translocation (Fig. 6A,B). HIF-1α and HIF-2α shRNA treatment reduced the protein levels of HIF-1α and HIF-2α in HPFs, respectively (Fig. 6C,D,E). Knockdown of HIF-2α, but not HIF-1α, also blocked the hypoxia-induced NFATc2 nuclear translocation (Fig. 6F,G).

Furthermore, we determined whether HIF-1α and HIF-2α could increase NFAT transcriptional activity. HEK 293 T cells were co-transfected with the NFAT reporter and either a HIF-1α or HIF-2α expression vector. Overexpression of HIF-2α significantly increased NFAT reporter activity, whereas HIF-1α overexpression had no effects on the NFAT reporter activity (Fig. 6H). Taken together, these results indicate that HIF-2α mediates the hypoxia-induced activation of NFATc2 signaling.

### NFAT signaling is involved in hypoxia-induced fibroblast proliferation

**Figure 7:**
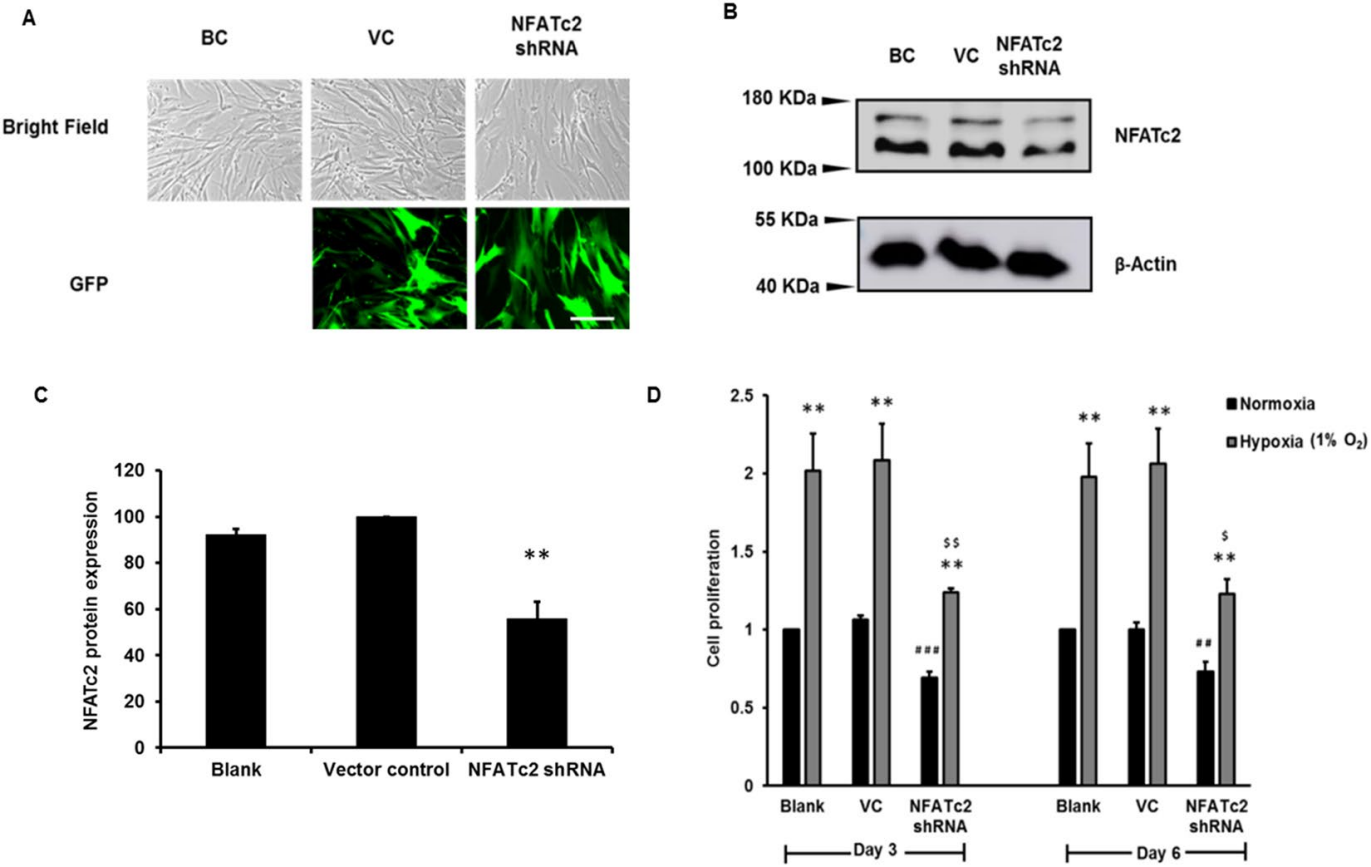
NFATc2 silencing inhibits hypoxia-induced cell proliferation. HPF cells were infected with a lentivirus expressing NFATc2 shRNA or vector control (VC) at an MOI of 100 and exposed to normoxia (21% O_2_) and hypoxia (1% O_2_) for 6 days. (**A**) Microscopic images of bright fields and green fluorescence protein (GFP) of blank control (BC), vector control (VC) and NFATc2 shRNA-treated cells. Scale Bar: 50 µm. (**B**) Western blot showing NFATc2 silencing. (**C**) Quantitation of NFATc2 levels was performed with ImageJ software. Data were normalized to VC. Values represent means ± SE. **p < 0.05 vs. VC. (**D**) Cell proliferation by BrdU assay. Cells were incubated with BrdU for 12 hrs. Data were normalized to the blank at normoxia. The absorbance values at normoxia were 0.09 ± 0.03 and 0.13 ± 0.01 for day 3 and day 6, respectively. Values represent means ± SE. **p < 0.01 vs. normoxia. ^##^p < 0.01, ^# # #^p < 0.001 vs VC at normoxia. ^$^p < 0.05, ^$$^p < 0.01 vs VC at hypoxia. n = 3 independent experiments.

To determine whether hypoxia-induced cell proliferation in lung fibroblasts occurs through the NFAT signaling pathway, NFATc2 was knocked down in HPF cells using a shRNA lentiviral vector expressing GFP. A high efficiency of lentiviral infection was observed (Fig. 7A). Western blot analysis showed that the NFATc2 protein level was reduced by 44 ± 7% in the NFATc2 shRNA-treated HPFs (Fig. 7B,C). NFATc2 knockdown significantly inhibited hypoxia-induced HPF proliferation (Fig. 7D). A significant decrease in HPF proliferation was also observed in NFATc2-knocked-down cells under normoxic conditions, likely due to the basal activity level of NFATc2. These results indicate that NFATc2 is a key regulator of cell proliferation in both normoxic and hypoxic conditions.

### Hypoxia-induced proliferation does not occur through upstream components of NFAT signaling

**Figure 8:**
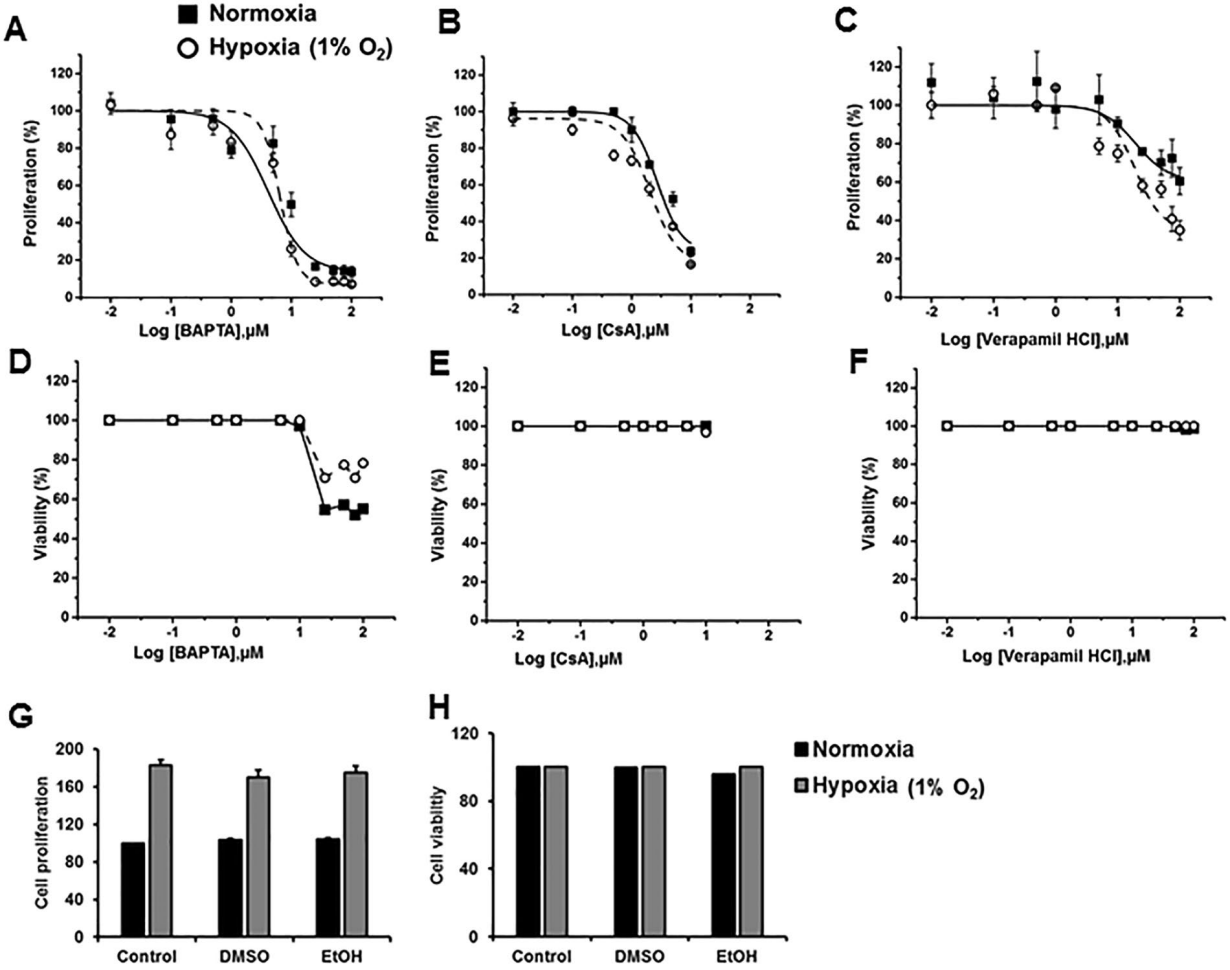
Effects of NFAT signaling inhibitors on hypoxia-induced proliferation. HPFs were treated with a calcium chelator (BAPTA-AM) and calcineurin inhibitor (cyclosporine A, CsA) and a calcium channel blocker (verapamil hydrochloride) and exposed to normoxia (21% O_2_) and hypoxia (1% O_2_) for 3 days. (**A**,**B** and **C**) Cell proliferation as determined by BrdU assay. Cells were incubated with BrdU for 12 hrs. (**D**,**E** and **F**) Cell viability as determined by LDH assay. (**G**,**H**) HPFs were treated with the solvents used [1% DMSO and 0.1% Ethanol (EtOH)] and exposed to normoxia (21% O_2_) and hypoxia (1% O_2_) for 3 days. Cell proliferation and cell viability were determined by BrdU assay and LDH assay, respectively. Data were expressed as a percentage of the control (0 µM inhibitor) at each oxygen condition. The absorbance of control at normoxia for proliferation was 0.39 ± 0.06 (n = 3) and for hypoxia was 0.66 ± 0.16 (n = 3). Viability % was calculated as 100% - cytotoxicity %. Cytotoxicity % = [(LDH activity of sample -Spontaneous LDH activity)/(Maximum LDH activity -Spontaneous LDH activity)] × 100. The absorbance of control at normoxia for LDH activity was 0.17 (n = 2) and for hypoxia was 0.16 (n = 2). Maximum and spontaneous LDH activity values for normoxia were 0.26 and 0.17 (n = 2), respectively. Maximum and spontaneous LDH activity values for hypoxia were 0.27 and 0.17 (n = 2), respectively. Values represent means ± SE for proliferation and means for viability.

Hypoxia activates voltage-gated Ca^2+^ channels (VGCCs)^14,33^. With the exception of NFAT5, all the other isoforms of the NFAT family are activated through calcium sensing. An increased intracellular calcium influx activates calcineurin, which dephosphorylates cytoplasmic NFAT and results in NFAT nuclear translocation, leading to the transcription of genes involved in cell proliferation^18,20^. To determine which step of NFAT signaling is involved in hypoxia-induced fibroblast proliferation, we inhibited NFAT signaling at various steps, including Ca^2+^ channels, intracellular Ca^2+^ and calcineurin, using specific inhibitors and determined cell proliferation. Verapamil is an L-type voltage-gated Ca^2+^ channel blocker that reduces Ca^2+^ influx^34,35^. BAPTA-AM is a specific Ca^2+^ chelator^36,37^. Cyclosporine A (CsA) is a calcineurin inhibitor that inhibits the enzymatic activity of calcineurin^38,39^. HPF cells were treated with different concentrations of these inhibitors and exposed to normoxia or hypoxia for 3 days. HPF cells treated with the Ca^2+^ chelator BAPTA-AM and the calcineurin inhibitor CsA did not show any differential effects in HPF proliferation between normoxic and hypoxic conditions (Fig. 8A–C). These results suggest that the upstream molecules of NFAT signaling prior to NFAT nuclear translocation are not involved in hypoxia-induced pulmonary fibroblast proliferation. However, treatment with the Ca^2+^ channel blocker verapamil chloride showed differential effects on cell proliferation between normoxic and hypoxic conditions at higher concentrations (Fig. 8C). This could be due to its direct effects on proteins involved in cell cycle and proliferation^40^. The inhibitors did not affect cell viability, except for BAPTA-AM, which was associated with cell death at concentrations greater than 10 µM (Fig. 8D–F).

Since we used dimethyl sulfoxide (DMSO) to dissolve TC7009, KC7F2 (Fig. 3), BAPTA, and CsA, and ethanol (EtOH) to dissolve verapamil chloride (Fig. 8), we examined whether these solvents had any effects on cell proliferation and cell viability. HPFs were treated with 1% DMSO and 0.1% ethanol, the maximum concentrations used in this study, and exposed to 1% O_2_ or normoxia for 3 days. These solvents at the concentrations used did not affect cell proliferation and viability (Fig. 8G,H).

## Discussion

**Figure 9:**
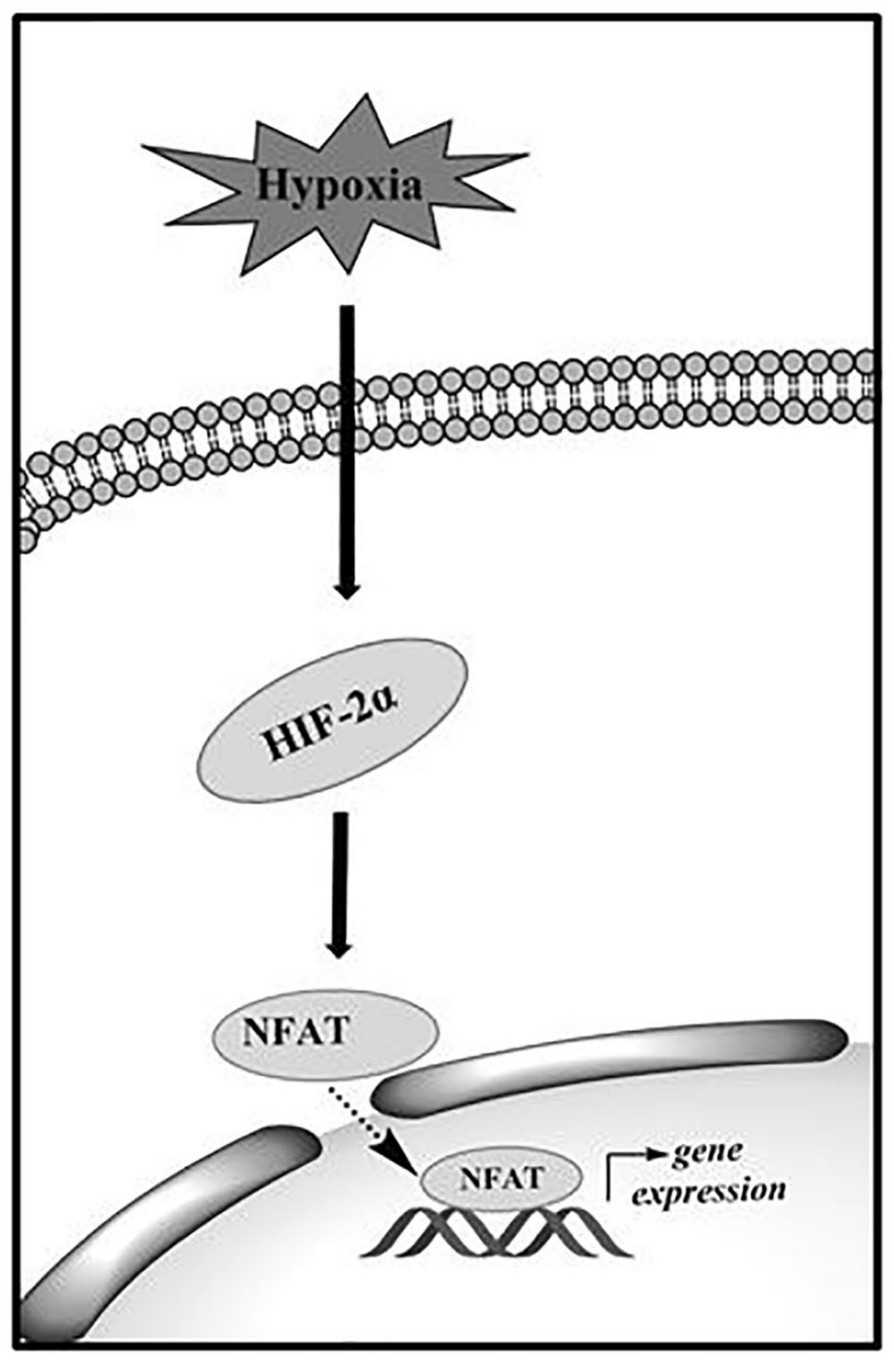
A proposed model of hypoxia-induced fibroblast proliferation through NFAT signaling. Hypoxia induces the HIF-2α expression in human pulmonary fibroblasts which activates the NFAT signaling. HIF-2α promotes the translocation of NFAT(c2) from cytoplasm to nucleus, which induces the expression of the genes responsible for cell proliferation.

Hypoxia regulates many cellular events, including growth, proliferation and apoptosis, in different cell types. In this study, we demonstrated that 1) hypoxia promotes normal and IPF human pulmonary fibroblast proliferation, 2) hypoxia promotes the G1/S phase transition, 3) hypoxia activates NFAT signaling via HIF-2α, and 4) NFATc2 is required for hypoxia-induced cell proliferation. Based on these results, we propose the following model (Fig. 9). Hypoxia activates NFATc2 signaling by promoting NFATc2 nuclear translocation and/or transcriptional activity via HIF-2α, which in turn induces gene expression related to cell proliferation.

Our FACS analysis indicated that hypoxia (1% O_2_) promoted the G1/S phase transition of pulmonary fibroblasts. This is consistent with several previous studies that have shown that moderate hypoxia (1–2% O_2_) accelerates the G1/S phase transition in Jurkats (human T lymphocytes), SUP-T1s (human T lymphoblasts)^41^, human mesenchymal stem cells^42^, keloid fibroblasts^43^ and normal human lung fibroblasts^13^. However, severe hypoxia (0.1–0.5% O_2_) has been shown to arrest the G1/S phase transition in adult human lung fibroblasts^13^, as well as rat and mouse embryonic fibroblasts^44^.

The cell cycle is regulated by cyclins and cyclin-dependent kinases (CDKs). Cyclin E promotes the G1/S phase transition and its expression increases at the G1/S checkpoint. Cyclin A promotes the G2/M phase transition^45,46^. Our Western blot analysis showed that hypoxia increased cyclin E1 and downregulated cyclin A2 protein levels, which is consistent with our finding that hypoxia increased S phase transition in human pulmonary fibroblasts. Upregulation of cyclin E1 levels by hypoxia has been observed in prostate cancer cells^47^. Cyclin E has also been shown to overcome G1 arrest in rat embryonic fibroblasts^48^.

Cyclin D1 expression is high in the G1 phase, and its expression is reduced when cells enter the S phase^45,49–51^. Cyclin D1 promotes cancer cell growth^52–54^. Unexpectedly, we observed downregulation of cyclin D1 levels in pulmonary fibroblasts with hypoxia. However, minimal nuclear-localized cyclin has been observed when BrdU incorporation is high in proliferating human lung fibroblasts^49,51,55^. An inverse correlation between BrdU incorporation and nuclear cyclin D1 levels has also been reported^51^.

CDK2 is mainly involved in regulating S and G2 phase, while CDK4 and 6 are primarily involved in the G1 phase^45^. We did not observe any changes in CDK2, 4 and 6 levels with hypoxia. This is consistent with the observation that CDK protein expression persists stably throughout the cell cycle, whereas cyclin expression varies^46^. However, CDK2 expression is reduced by severe hypoxia (0.1%-0.5% O_2_)^44^ in healthy lung fibroblasts, but increased by hypoxia in prostate cancer cells^49^. Moderate hypoxia (2% O_2_) has also been shown to inhibit CDK inhibitor p21^13^.

The involvement of HIF isoforms varies in different cellular mechanisms associated with proliferative responses. Our inhibitory study demonstrated that a HIF-2α inhibitor, but not a HIF-1α inhibitor, decreased the hypoxia-mediated proliferation in HPFs, suggesting that hypoxia-mediated pulmonary fibroblast proliferation is HIF-2α-dependent. This finding was supported by knockdown experiments in which the knockdown of HIF-2α in IPF fibroblasts reduced cell proliferation under hypoxic conditions (3% O_2_)^11^. Furthermore, pulmonary arterial fibroblast proliferation is decreased by the knockdown of HIF-2α, but not HIF-1α, under moderate hypoxic conditions (1% O_2_)^56^. Interestingly, HIF-2α promotes cell proliferation in renal-cell adenocarcinoma cells (WT8, 786 –O RCC), whereas HIF-1α inhibits the proliferation of HCT116 colon carcinoma cells at 0.5% O_2_^9^.

The mechanisms underlying the increase in fibroblast proliferation in response to hypoxia may be complex and involve several pathways. It has been reported that hypoxia increases IPF fibroblast proliferation by inducing miR-210 expression, which in turn reduces the c-myc inhibitor MNT^11^. Hypoxia-induced increases in miR-210 level is in a HIF-2α-dependent fashion and appears early and persists through day 6. In the current study, we propose that hypoxia increases fibroblast proliferation by activating NFATc2 signaling via HIF-2α, which in turn regulates cell cycle protein expression. The activation of NFATc2 signaling by hypoxia is supported by our observations that hypoxia promotes NFATc2 nuclear translocation at an early time point (day 3) and increases NFATc2 mRNA levels at a late time point (day 6). It is conceivable that effects of HIF-2 on fibroblast proliferation may be mediated by several pathways - an early pathway through miR-210 and NFATc2 nuclear translocation and later effects involving NFATc2 transcriptional activity.

The nuclear translocation of NFAT isoforms caused by hypoxia has been previously reported in other cells. Increased NFATc3 nuclear translocation has been observed in PASMCs of mice that have been subjected to chronic hypoxic conditions^21^. In another study, exposure of human PASMCs from PAH to hypoxia for 96 hrs resulted in increased NFATc2 nuclear signal compared to normoxic conditions^17^. Neural stem cells that are exposed to hypoxia showed increased NFATc4 nuclear localization^28^. In an ischemia and reperfusion kidney model, rat IMCD (inner renal medulla Sprague-Dawley) cells exposed to anoxia (O_2_ replaced by N_2_) for 2 hrs had higher NFAT5 nuclear signals compared to cells exposed to normoxia^57^. U373MG human glioblastoma astrocytoma cells treated with the hypoxia-mimicking agent CoCl_2_ had increased NFAT nuclear localization^58^. PASMCs isolated from mice exposed to chronic 21-day hypoxia showed higher NFAT activity compared to those from normoxic mice^59^.

Hypoxia-mediated activation of NFATc2 occurs through HIF-2α, not HIF-1α, as knock-down or chemical inhibition of HIF-2α, not HIF-1α, inhibited hypoxia-induced NFATc2 nuclear translocation, and overexpression of HIF-2α, not HIF-1α, increased NFATc2 transcriptional activity. There are two possibilities by which HIF-2α may activate NFATc2 signaling. One possibility is that HIF-2α induces the expression of positive components in NFAT signaling or the genes that upregulate NFAT signaling. Indeed, our study showed that hypoxia increases NFATc2 expression in HPFs. It has also been shown that the activation of HIF-2α increases NFATc2 and c3 mRNA expression levels^60,61^. Another possibility by which HIF-2α may activate NFAT signaling is that HIF-2α directly interacts with NFATc2.

Our recent studies showed that NFATc2 is required for Wnt5a-mediated cell proliferation^31^. Our current studies indicate that the silencing of NFATc2 reduced the fibroblast proliferation in hypoxic conditions, suggesting that NFATc2 signaling is also involved in hypoxia-mediated fibroblast proliferation. Different isoforms of NFAT have been reported to be involved in the regulation of cell proliferation in different cell types^25–28,62^. In pulmonary arterial hypertension (PAH), PASMCs show an increased proliferation and resistance to apoptosis. A chemical treatment (Plumbagin), which inhibits NFATc2 gene expression and NFATc2 nuclear localization, resulted in decreased cell proliferation of PASMCs derived from PAH patients, suggesting a positive relationship between NFATc2 signaling and cell proliferation of PASMCs in PAH^27^. Serotonin treatment in PASMCs increases cell proliferation and activates calcineurin and NFAT signaling. The inhibition of calcineurin by CsA and silencing of NFATc2 by siRNA decreases DNA synthesis in PASMCs treated with serotonin, indicating that NFATc2 is involved in proliferation^25^. In mice, conditional expression of NFATc1 in β-cells promoted β-cell proliferation^62^. In another study, silencing of NFATc4 in mouse brains through in utero electroporation of siRNA reduced the number of cells in the S phase of the cell cycle of neural progenitor cells and the hypoxia-mediated neurosphere progenitor forming capacity in neural stem cells, indicating the involvement of NFAT signaling in cell proliferation^28^.

Hypoxia activates voltage-gated Ca^2+^ channels (VGCC)^14,33^, which in turn may activate NFAT signaling and thus cell proliferation. However, in our studies, inhibiting the upstream intracellular Ca^2+^ and calcineurin molecules in NFAT signaling did not differentially affect cell proliferation under normoxic and hypoxic conditions, although treatment with a Ca^2+^ channel blocker did inhibit cell proliferation more efficiently under hypoxic conditions than normoxic conditions. A direct interaction of calcium channels and cell cycle proteins and kinases has previously been reported^40^. Thus, the observed effects of the Ca^2+^ channel blocker on fibroblast proliferation may be due to the direct interaction of cell cycle proteins and kinases with the Ca^2+^ channels.

In summary, we conclude that hypoxia-induced cell proliferation occurs via the activation of NFAT signaling through HIF-2α.

## Methods

### Cell culture

Primary human pulmonary fibroblasts (HPFs) isolated from a 74-year-old male Caucasian subject were purchased from PromoCell (Heidelberg, Germany, Cat. No: C-12361). They were cultured in Fibroblast Basal Medium (PromoCell, Cat. No: C-23220) with its recommended supplements (PromoCell, Cat. No: C-39320), including 0.02 ml/ml fetal calf serum, 1 ng/ml basic fibroblast growth factor and 5 µg/ml insulin. IPF fibroblasts LL29 and LL97A were isolated from a 26-year-old female Caucasian patient and a 48-year-old male Caucasian patient, respectively, and were purchased from American Type Culture Collection (ATCC, Manassas, VA). Human normal lung fibroblasts CCD-13Lu and CCD-19Lu were isolated from a 71-year-old male Black patient with carcinoma and a 20-year-old Caucasian female, respectively, and were also purchased from ATCC. Human normal lung fibroblasts HLF153 and HLF154 and IPF fibroblasts IPF12 and IPF14 were isolated as previously described^11^. All of these cells were maintained in F12K medium containing 10% fetal bovine serum and 1% penicillin-streptomycin. Human embryonic kidney (HEK) epithelial 293 T cells were purchased from ATCC and maintained in DMEM containing 10% fetal bovine serum and 1% penicillin-streptomycin.

### Hypoxia exposure

Cells were exposed to normoxia (21% O_2_) in a normal CO_2_ cell culture incubator or hypoxia (5% or 1% O_2_) in a hypoxia chamber (Billups-rothenburg, Del Mar, CA). Sterile water was placed inside the chamber to maintain moist conditions. Hypoxic conditions were created by filling the chamber with 5% or 1% O_2_, 5% CO_2_ and balanced N_2_. The gas was passed through the chamber at 1–2 psi for 3 min, and the chamber was sealed and placed in a 37 °C cell culture incubator. The oxygen concentration in the chamber was monitored using an oxygen sensor (OxyCheq, Marianna, FL). Every 2 days, media were replaced with fresh media that were degassed using a SpeedVac Concentrator system (Thermo Fisher Scientific, Waltham, MA).

### Cell proliferation assay

Cell proliferation was determined using a BrdU assay (EMD Millipore, St Charles, MO), which measures the incorporation of bromodeoxyuridine (BrdU), a thymidine analog of DNA. Fibroblasts were seeded at a density of 2,000 cells/well in 96-well plates. After 24 hrs, the medium was replaced with fresh medium and incubated under normoxic or hypoxic conditions for 3 or 6 days. Then, 3–12 hrs prior to the endpoint, BrdU was added and cells were fixed with fixing solution provided by the BrdU assay kit manufacturer. A BrdU detection antibody was added and incubated for 1 hr at room temperature. After washing, peroxidase-labeled goat anti-mouse IgG was added and incubated for 30 min at room temperature. After a final wash, substrate was added and incubated for 30 min in room temperature at dark. Then, stop solution was added, and the absorbance was measured at 450 nm using a Spectramax M2 microplate spectrophotometer (Molecular Devices, Sunnyvale, CA).

### Cell viability

Cell viability was determined using an MTT assay kit (ATCC, Manassas, VA) or LDH assay (Thermo Scientific, Waltham, MA). Briefly, cells were cultured in 96-well plates at a density of 2,000 cells/well and exposed to normoxia or hypoxia for 3 or 6 days. For MTT assays, 10 µL of MTT reagent was added to the cells. After 4 hrs of incubation, 100 µL of detergent reagent was added. Plates were maintained at room temperature in the dark for 2 hrs, and the absorbance values were subsequently recorded at 570 nm. For LDH assay, 50 µL of the supernatant was transferred to new 96-well plates. Then, 50 µL of the reaction mixture (combination of substrate and assay buffer) was added to the supernatant, and plates were incubated at room temperature in the dark. After 30 min, 50 µL of stop solution was added, and the absorbance was measured at 490 nm and 680 nm. Absorbance values at 680 nm (background) were subtracted from absorbance values at 490 nm to obtain LDH activity.

### Cell cycle assay

Incorporation of propidium iodide in each phase of the cell cycle was analyzed by fluorescence-activated cell sorting **(**FACS), using a cell cycle phase determination kit (Cayman, Ann Arbor, MI). Briefly, HPF cells were seeded at a density of 100,000 cells/well in 6-well plates and serum-starved for 24 hrs. The following day, fresh medium with serum was added, and the cells were exposed to normoxic and hypoxic (1% O_2_) conditions for 3 days. The cells were trypsinized, counted, fixed and analyzed according to the manufacturer’s instructions.

### Inhibitor studies

HPF cells were seeded at a density of 2,000 cells/well in 96-well plates. Then, 24 hrs after seeding, the medium was replaced with fresh medium with and without the following inhibitors at concentrations ranging from 0–100 µM: HIF-1α inhibitor KC7F2 (TOCRIS, Pittsburg, PA), HIF-2α inhibitor TC-S 7009 (TOCRIS, Pittsburg, PA), calcium chelator BAPTA-AM (Life Technologies, Carlsbad, CA), calcium channel blocker verapamil chloride (Sigma, St. Louis, MO), and calcineurin inhibitor cyclosporine A (CsA, R&D Systems, Minneapolis, MN). Cells were treated with these compounds and exposed to normoxic (21% O_2_) or hypoxic conditions (1% O_2_) for 3 days in the presence of the inhibitors for the entire 3 days. Separate sets of wells were maintained for media and solvent controls. Cell proliferation and cell viability were determined by BrdU assay (EMD Millipore, St Charles, MO) and LDH assay (Thermo Scientific, Waltham, MA), respectively. Cell proliferation and viability graphs were plotted using Origin (OriginLab, Northampton, MA) software with DoseResp function.

### Quantitative real-time PCR

Fibroblasts were seeded at a density of 35,000 cells/well in 6-well plates and incubated at 37 °C overnight. The following day, after replacing the media with fresh media, cells were exposed to normoxia and hypoxia for 3 and 6 days. Total RNA was extracted using TRIzol Reagent (Molecular Research Center, Cincinnati, OH). One microgram of DNase-treated RNA was used for first-strand cDNA synthesis using random primers and Moloney Murine Leukemia Virus reverse transcriptase (Thermo Fisher Scientific, Waltham, MA). cDNA was diluted 100 times and then used for quantitative real-time PCR analysis using SYBR green (Eurogentec, Fremont, CA). β-actin was used as the reference gene. Forward and reverse primers for human NFTAc1-c4, NFAT5, and β-actin are listed in Table 1. Relative gene expression was calculated as 2^−ΔCt^ = 2^−(target Ct-reference Ct)^.

**Table 1:** Primers used for real-time PCR.

human-NFATC1-FW	CCGTATGAGCTTCGGATTGAG
human-NFATC1-RE	TCATTCTCCAAGTAGCCATGCA
human-NFATC2-FW	CGGGCCCACTATGAGACAGA
human-NFATC2-RE	CCAGAGGCTTGTTTTCCATGTAG
human-NFATC3-FW	TCGAGCCCATTATGAAACTGAA
human-NFATC3-RE	GATCATCTGCTGTCCCAATAAACA
human-NFATC4-FW	GTGGCTGGCATGGACTACCT
human-NFATC4-RE	AGCTCCAGCTGCTCATATTGG
human-NFAT5-FW	AGTGCCAAAGCACCTCACTATG
human-NFAT5-RE	CCAGTTCCTTTTTGGTTTTCCA
human-β-actin -FW	GCCGGGACCTGACTGACTAC
human-β-actin-RE	TTCTCCTTAATGTCACGCACGAT

FW: Forward, RE: Reverse.

### NFAT Reporter Assay

HEK 293 T cells were seeded at a density of 20,000 cells/well in 96-well plates. The following day, cells were transfected with 70 ng of NFAT–firefly reporter plasmid pGL4.30 [*luc2P*/NFAT-RE/Hygro] Vector (Cat.No: E8481, Promega, Madison, WI), which has a minimal promoter and an NFAT response element, 2 ng of pRL-TK (*Renilla*) (Promega, Madison, WI) internal control plasmid, and HIF-1α expression vector (Addgene, Cambridge, MA, Cat. No: 18949), 40 ng of HIF-2α expression vector (Addgene, Cat. No: 18950) or a control plasmid, PCMV (Origene, Rockville, MD. Cat.No: PCMV6-XL4) for 24 hrs with Lipofectamine 2000 (Thermo Fisher Scientific, Waltham, MA). The next day, cells were washed with serum-free medium, and fresh medium was added. After 24 hrs, dual luciferase activities were measured using a Dual-Luciferase® Reporter Assay System (Promega, Madison, WI). The results were expressed as the ratio of firefly to *Renilla* luciferase activities.

### Western blot

To analyze NFATc2 levels, HPF cells were cultured in 6-well plates at a density of 35,000 cells/well and exposed to normoxia and hypoxia for 6 days. Proteins were extracted with RIPA buffer (Cell Signaling, Beverly, MA) containing 1X phosphatase and protease inhibitors (Thermo Fisher Scientific, Waltham, MA). Cell debris was removed by centrifugation (20,000 × g for 15 min) and supernatants were collected. Protein concentration was determined using a Bio-Rad (Hercules, CA) protein assay kit. Fifty-five µg of proteins were separated on 8% SDS PAGE gels for detecting NFTAc2 expression.

For detecting cyclin, cyclin-dependent kinases (CDKs), HIF-1α, and HIF-2α, cells were grown at a density of 0.05–0.10 × 10^6^ cells/well in 6 well plates and exposed to normoxia and hypoxia for 3 days. Whole cell lysates were extracted using a buffer containing 70% (v/v) 0.5 M Tris (pH 6.8), 12.8% (w/v) SDS, 30% (v/v) glycerol, 6% (v/v) 2-mercapto-ethanol and 0.012% (w/v) bromophenol blue. Similar amounts of cell lysates were separated on 10% SDS PAGE gels.

After being transferred to the membranes, the blots were blocked with 5% non-fat milk in Tris-Buffered Saline with Tween^®^20 (TBST) buffer. The following antibodies were added, and membranes were incubated at 4 °C overnight: polyclonal rabbit anti-NFATc2 (1:200 dilution, Santa Cruz Biotechnology, Santa Cruz, CA, Cat. No: 13034), monoclonal mouse anti-cyclin A2 (1:1000 dilution, Cell signaling, Beverly, MA, Cat. No: 4656), monoclonal mouse anti-cyclin D1 (1:750 dilution, Santa Cruz Biotechnology, Cat. No: 8396), monoclonal mouse anti-cyclin E1 (1:1000 dilution, Cell signaling, Cat. No: 4129), polyclonal rabbit anti-CDK2 (1:750 dilution, Santa Cruz Biotechnology, Cat. No: 163), polyclonal rabbit anti-CDK4 (1:750 dilution, Santa Cruz Biotechnology, Cat. No: 260), polyclonal rabbit anti-CDK6 (1:750 dilution, Santa Cruz Biotechnology, Cat. No: 177), monoclonal mouse anti-HIF-1α (1:300 dilution, BD biosciences, La Jolla, CA, Cat. No: 610958), polyclonal rabbit anti-HIF-2α (1:500 dilution, Novus biologicals, Littleton, CO, Cat. No: 100-122), and monoclonal mouse anti-actin (β-actin) (1:2000 -1:5000 dilutions, Thermo Fisher Scientific, Waltham, MA, Cat. No: MA5-15739). Horseradish peroxidase-conjugated goat anti-rabbit or goat anti-mouse secondary antibodies (Jackson Immunoresearch, West Grove, PA,) were added at a dilution of 1:2000-1:3000, and the membranes were incubated at room temperature for 1 hr. Then, blots were developed by adding the super signal substrate (Thermo Fisher Scientific, Waltham, MA), and images were obtained with an Amersham Imager 600 (GE Healthcare, Pittsburg, PA).

### shRNA lentivirus construction

NFATc2, HIF-1α and HIF-2α shRNAs were designed by BLOCK-iT™ RNAi Designer software from Invitrogen (Grand Island, NY). The sequence for NFATc2 shRNA is GGGATCTTGAAGCTTAGAAAC [human NFATc2, NM_012340, position in coding DNA sequence (CDS) 1549–1569]. The sequence for HIF-1α shRNA is GCTGATTTGTGAACCCATTCC [human HIF-1 α NM_001530.3, position in coding DNA sequence (CDS) 1067–1087]. The sequence for HIF-2α shRNA is GCTTCAGTGCCATGACAAACA [human HIF-2α NM_001430.4, position in coding DNA sequence (CDS) 2198–2218]. These shRNA fragments were inserted into the pSIH-H1 plasmid vector (System Biosciences, Mountain View, CA) as described previously^63^. For the 5′ end, which is the BamH1 restriction enzyme site, a sequence of GATCC was added to the shRNA target sequence. Then, a TTCAAGAGA loop sequence was added. Finally, to the 3′ end, the antisense sequence of the above target sequence (i.e., for NFATC2 shRNA, GTTTCTAAGCTTCAAGATAAA followed by TTTTTG) was added. This complete sequence was considered as the forward oligo. For the reverse oligo, at the 5′ end EcoRI restriction enzyme site, AATTCAAAA was added. Then, the loop sequence TCTCTTGAA followed by antisense sequence of GTTTCTAAGCTTCAAGATAAA was added. Finally, one G was added to the 3′ end of the reverse oligo. These forward and reverse oligos were mixed with annealing buffer (Promega, Madison, WI), and the mixture was heated to 90 °C for 4 minutes followed by 37 °C for 15 minutes. The pSIH-H1 vector (System Biosciences, Mountain View, CA) was double-digested with BamHI and EcoRI restriction enzymes. Then, the annealed product was ligated to the pSIH-H1 plasmid vector (System Biosciences, Mountain View, CA) using T4 ligase (Promega, Madison, WI). A control vector containing scrambled shRNA was purchased from System Biosciences (Mountain View, CA, Cat.No: SI505A-1). Both plasmids were used to transform *E*. *coli*–ST3 competent cells and amplified. Lentiviruses containing the shRNA were produced in HEK 293 T cells, and virus titers were determined as described previously^63,64^. In brief, Lenti-X™ HTX Packaging vectors (Clontech, Mountain View, CA) were used for preparing lentiviruses in HEK 293 T cells. For titer determination, viruses were serially diluted and used to infect HEK 293 T cells in 12-well plates. Then, green fluorescent protein (GFP)-positive cells were counted at 20 × magnification under a fluorescence microscope in 10–15 fields per well. For titer calculation, the average number of GFP–positive cells was multiplied by 594 (which is the number of fields in a well of a 12-well plate at 20X magnification) and divided by the volume of the medium in the well and the virus dilution.

### Effect of NFATc2 silencing on hypoxia-mediated cell proliferation

HPF cells were seeded at a density of 2,000 cells/well in 96-well plates. Cells were infected with a lentivirus containing either vector control or NFATc2 shRNA at a multiplicity of infection (MOI) of 100 using a fibroblast growth medium containing polybrene (8 µg/mL, EMD Millipore, St Charles, MO). After 24 hrs, cells were examined under a fluorescence microscope for GFP to determine the infection efficiency. Then, the virus was removed and fresh media was added. The infected cells were exposed to normoxic and hypoxic conditions (1% O_2_) for 3 and 6 days. Cell proliferation was measured using BrdU assay (EMD Millipore, St Charles, MO).

### Immunofluorescence staining

HPF cells were cultured at 2,000 cells/well in 8-well glass chamber slides (EMD Millipore, St Charles, MO) and exposed to normoxia (21% O_2_) and hypoxia (1% O_2_) at 37 °C for 3 days. For positive controls, cells were treated with ionomycin (Sigma-Aldrich, St. Louis, MO,) at a concentration of 1 μg/mL for 24 hrs. For silencing HIF-1α and HIF-2α, the cells were infected with a shRNA lentivirus at an MOI of 100 for 24 hrs. After adding fresh media, cells were exposed to normoxia and hypoxia for 3 days. After incubation, cells were fixed with 10% formalin and permeabilized with 0.1% Trition-X-100. Rabbit polyclonal anti-NFATc2 antibodies (Santa Cruz biotechnology, Santa Cruz, CA) were added at a dilution of 1:50 and incubated at 37 °C for 1 hr. Alexa Flour 488- or 546-conjugated goat anti-rabbit secondary antibody (Life technologies, Carlsbad, CA) was added at a 1:300 dilution and incubated for 1 hr at 37 °C Nuclei were stained with Hoechst 33342 (Molecular probes, Waltham, MA) at a final concentration of 5 µg/mL. Images were captured by a digital camera and MetaVue software (Universal Imaging Co., Downingtown, PA) under a fluorescence microscope (Nikon, Melville, NY). Fields were randomly selected, and five images were taken for each condition. Nuclear NFATc2-positive cells and the total number of cells were counted in each field. NFATc2 nuclear-translocated cells were calculated as the percentage of nuclear NFATc2-positive cells relative to the total number of cells.

### Statistical analysis

The data were presented as the means ± SE. Statistical analysis was performed by Student’s t-test for two groups or one-way ANOVA, followed by Tukey’s multiple comparisons test, for multiple groups using SPSS 16.0 or GraphPad Prism 7 software. p < 0.05 was considered statistically significant.
